# A preliminary study of diffusion tensor imaging in root entry zone of primary trigeminal neuralgia

**DOI:** 10.3389/fnana.2023.1112662

**Published:** 2023-03-22

**Authors:** Yiwen Wang, Danni Wang, Yingwei Wu, Ce Zhu, Wenbin Wei, Yao Li, Lingzhi Li, Wantao Chen, Minjie Chen

**Affiliations:** ^1^Department of Oral Surgery, Ninth People’s Hospital, Shanghai Jiao Tong University School of Medicine, Shanghai, China; ^2^College of Stomatology, Shanghai Jiao Tong University, Shanghai, China; ^3^National Center for Stomatology, Shanghai, China; ^4^National Clinical Research Center for Oral Diseases, Shanghai, China; ^5^Shanghai Key Laboratory of Stomatology, Shanghai, China; ^6^School of Biomedical Engineering, Shanghai Jiao Tong University, Shanghai, China; ^7^Department of Radiology, Ninth People’s Hospital, Shanghai Jiao Tong University School of Medicine, Shanghai, China; ^8^Department of Preventive Dentistry, Ninth People’s Hospital, Shanghai Jiao Tong University School of Medicine, Shanghai, China; ^9^Department of Stomatology, Huashan Hospital, Fudan University, Shanghai, China; ^10^Department of Oral and Maxillofacial-Head & Neck Oncology, Shanghai Ninth People’s Hospital, Shanghai Jiao Tong University School of Medicine, Shanghai, China

**Keywords:** primary trigeminal neuralgia, magnetic resonance imaging, diffusion tensor imaging, root entry zone, neurovascular compression (NVC)

## Abstract

**Objective:**

Primary Trigeminal Neuralgia (PTN) is a common and refractory neurological disease. Conventional vascular compression theory could not completely explain the etiology and pathogenesis of PTN. This study used diffusion tensor imaging (DTI) to demonstrate the microstructural changes of root entry zone (REZ) region in PTN patients.

**Materials and methods:**

DTI sequences was performed on PTN patients and healthy controls (HCs). Clinical data included affected side, disease course and visual analogue scale (VAS) were collected. Quantitative DTI variables such as FA, MD, AD and RD of the root entry/Exit zone (REZ) were measured and compared in PTN/HCs, affected/unaffected side, and pre/post operation groups. The PCoA was established to conduct overall differences between PTN group and the HCs.

**Results:**

A total of 17 patients with PTN (mean age 59.29 ± 8.53; 5 men) and 34 HCs (mean age 57.70 ± 6.37; 10 men) were included. Lower FA value of the affected side of PTN group was observed compared to the unaffected side and the HCs (*p* = 0.001), whereas the values of MD, AD and RD were significantly increased (*p* < 0.001). Moreover, the decrease of FA value was recovered post operation. PCoA results of the comprehensive indexes can significantly distinguish PTN group from HCs (*r* = 0.500, *p* < 0.001).

**Conclusion:**

Quantitative variables derived from DTI in REZ had significantly different profiles between PTN patients and HCs, which were associated with VAS situation and the disease course of PTN. The comprehensive index established on DTI variables were of great potential to reveal the microstructure changes in PTN patients and predict the therapeutic effect.

## 1. Introduction

Trigeminal neuralgia (TN) is the most common neuropathic pain that occurs in the oral and maxillofacial region. TN is defined as a recurrent facial pain that occurs in one or more branches of the trigeminal nerve; unilateral onset, not extending beyond the distribution of the trigeminal nerve, pain with the following characteristics such as pain lasting from a few seconds to two minutes; severe pain; pain with electric shock, shooting, knife-cutting, or sharp features; pain is evoked by innocuous stimuli within the affected trigeminal distribution and no other better diagnostic method exists (Katusic et al., 1990; MacDonald et al., 2000; Mueller et al., 2011; Headache Classification Committee of the International Headache Society (IHS), 2018; Cruccu et al., 2020). The International Classification of Headache Disorders, 3rd Editon (ICHD-3) in 2018 divides trigeminal neuralgia into three types: idiopathic, classic, and secondary TN. The idiopathic and classic TN were primary trigeminal neuralgia (PTN) in ICHD-2. Classic TN refers to trigeminal neuralgia caused by neurovascular compression (NVC) in cerebellopontine angle in MRI; idiopathic TN refers to trigeminal neuralgia with no obvious abnormality in electrophysiological tests and MRI without NVC. PTN patients have the characteristic clinical manifestations in the above definition (Maarbjerg et al., 2017; Headache Classification Committee of the International Headache Society (IHS), 2018).

The pathogenesis of PTN is still unclear and controversial. The main etiology theories for PTN patients including peripheral etiology, central etiology, allergic reaction, viral infection, comprehensive etiology and so on. It is traditionally believed that PTN is caused by the injury of the peripheral trigeminal nerve. The neurovascular compression (NVC) theory, as a kind of peripheral etiology theory, is the mainstream etiology theory of PTN that is widely accepted. The nerve fiber in root entry/exit zone (REZ) of trigeminal nerve is a bare area that is several millimeters long and is not surrounded by myelin sheaths, so the nerves in this area are more likely to be contacted or compressed by pulsatile blood vessels, resulting in pain (Gardner, 1962; Gubian and Rosahl, 2017).

At present, the diagnosis of PTN still relies on the patients’ subjective symptom description. There is no obvious abnormality in the basic examination of the nervous system of PTN patients (Sjaastad and Bakketeig, 2007; Cruccu et al., 2016). Magnetic resonance tomography angiography (MRTA) is widely used to show whether there is obvious vascular compression of the trigeminal nerve in the cerebellopontine angle area. However, MRTA can just show structure changes – NVC, and the functional changes were not developed. As a result, some confusion arises: the presence of NVC does not necessarily result in PTN; about 24% of the normal population also has NVC of the cerebellopontine angle without pain is that it only shows structure changes but without functional changes (Leal et al., 2010, 2011).

In recent years, with the continuous development of imaging technology, diffusion tensor imaging technology (DTI) has greatly promoted the understanding of brain structure and functional cognition with central nervous diseases. DTI is the only non-invasive method currently used to determine the direction of living biological nerve fibers. It uses the different diffusion characteristics of water molecules in the body to provide more details of brain tissue, especially white matter (Lummel et al., 2015). According to the data information in the DTI image, it can provide the microstructure and information of the local tissue. At present, DTI has been widely used in the study of various neuropsychiatric diseases and can also be used as a navigation technology in brain surgery. The DTI research on PTN is currently mainly based on the whole brain research, and there are few studies on the local microstructural changes of the trigeminal nerve root.

This study intends to quantitatively analyze the local DTI diffusion values in the REZ of the trigeminal nerve in PTN patients and healthy control volunteers, and to find the specific microstructural changes. Subgroup analysis of patients were performed to evaluate the correlation between clinical data and DTI parameters.

## 2. Materials and methods

This trial recruited a total of 17 patients (12 females and 5 males, with an average age of 59.3 years) who were diagnosed with primary trigeminal neuralgia (PTN) from August 2018 to August 2020 in the Department of Oral Surgery of the Ninth People’s Hospital Affiliated to Shanghai Jiao Tong University School of Medicine. The diagnosis of PTN was made by two senior oral surgeons with extensive experience in the diagnosis of trigeminal neuralgia.

The inclusion criteria for patients were as follows: ①according to the diagnostic criteria of the International Classification of Headache Disorders (ICHD-3) (Headache Classification Committee of the International Headache Society (IHS), 2018); ②unilateral disease; ③right-handed; ④disease duration longer than 6 months; ⑤no previous history of cranial and maxillofacial surgery or radiotherapy and chemotherapy; ⑥disease-related drugs (such as carbamazepine tablets, etc.) were discontinued for more than 48 h.

The exclusion criteria for patients were as follows: ①patients with other chronic pain besides PTN; ②blood pressure over than 180/100 mmHg; ③fasting blood glucose over than 8 mmol/L; ④history of brain trauma or craniocerebral surgery, etc.; ⑤metal implants in the body; ⑥other mental or neurological diseases and claustrophobia.

The study also recruited 34 healthy subjects as the health control group (HC) (24 females and 10 males, with an average age of 57.7 years), matched with the PTN group in terms of average age, gender, handedness, etc. The exclusion criteria for HC were as same as those for PTN group.

The content of this trial complies with the Declaration of Helsinki (as revised in 2013), and all subjects voluntarily participated in this trial and signed the consent form. This trial was approved by the Ethics Committee of the Ninth People’s Hospital Affiliated to Shanghai Jiao Tong University School of Medicine.

### 2.1. Collection of clinical data of research subjects

Collect the basic clinical information of patients, including name, gender, age, course of disease, related medication history, affected side, affected branch, etc. All subjects underwent visual analogue scale (VAS, 0–10): 0 means no pain; 2–3 means mild tolerable pain; 4–6 means that the pain affects daily life and is still tolerable; 7–10 means that the pain is severe and affect life seriously. All subjects underwent MRTA examination of trigeminal nerve, and the NVC situation between the affected side and the contralateral side in the REZ was recorded. For the subjects in the HC group, the basic clinical information was collected as above, and VAS was performed.

### 2.2. Imaging sequence and parameters

All subjects removed all metal foreign objects on their bodies before the examination, fixed the subjects’ head with a sponge washer, instructed them to keep still and try not to move, stay awake, and close their eyes, breathe quietly, and wear earplugs for noise reduction before the examination. SIEMENS MAGNETOM Verio 3.0T MR scanning instrument and standard 8-channel head receiver coil for scanning were used to acquire the DTI data of all subjects (located in the Ninth People’s Hospital affiliated to Shanghai Jiao Tong University School of Medicine). The scanning range includes the entire frontal and temporal lobes of the subjects, most of the occipital and parietal lobes, and the scanning layers require a line parallel to the anterior and posterior symphysis. DTI images were acquired using a diffusion-weighted spin-echo sequence, and the scan parameters were as follows: repetition time (TR)/echo time (TE) = 8,400/85 ms, voxel size = 2.4 mm × 2.4 mm × 2.5 mm, field of view (FOV) = 230 mm, flip angle (FA) = 90°, slices = 45 and slice thickness = 2.5 mm, one non-diffusion volume (*b* = 0 s/mm^2^) and 29 diffusion volumes were collected (*b* = 1,200 s/mm^2^).

### 2.3. DTI data processing and analysis

Using dcm2nii.exe software to convert the data from DICOM to nifti format. Data preprocessing was performed with the FSL software package (FMRIB Software Library v6.0, Created by the Analysis Group, FMRIB, Oxford, UK).^1^ The images were performed by head motion and eddy current correction, non-brain tissue removal, fitting the diffusion tensor model to the corrected diffusion data through DTIFIT in FMRIB Diffusion Toolbox (FDT), and created the individual FA and Non-FA (AD, MD, and RD) images. Using DSI Studio software^2^ to manually reconstruct the trigeminal nerve fiber bundle. Four diffusion values of each subject from bilateral trigeminal REZ areas were measured: fractional anisotropy (FA), radial diffusivity (RD), axial diffusivity (AD), and mean diffusivity (MD).

**FIGURE 1:**
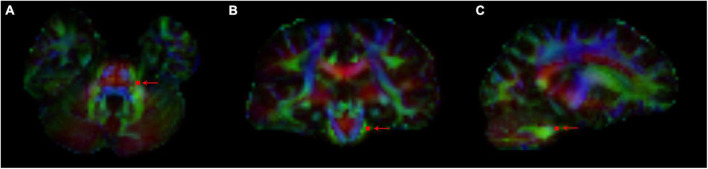
The path of trigeminal nerve fiber bundles was reconstructed on the generated color direction map, and ROI points were depicted (arrow indicated) in REZ. FA, RD, AD, and MD values of trigeminal nerve corresponding to ROI points in each REZ region were measured. **(A)** Horizontal position; **(B)** coronal position; and **(C)** sagittal position.

We generated the color direction map by superimposing the principal eigenvector map on the FA map and identified the trigeminal nerve in the horizontal, coronal and sagittal plane and can be clearly seen in the pontine-cisternal space in each subject. A region of interest (ROI) of 4 size voxel units was manually placed on each REZ in the REZ (Figure 1). And then we measured the value of FA, RD, AD and MD of each ROI. The values of each area were measured twice, and the average was taken, and the values of the four diffusion values in the HC group were taken as the average of the measured values on bilateral sides.

One-way ANOVA was used to compare the measured values of the REZ of the patients’ affected side, the patients’ unaffected side and the HC group; the least significant difference (LSD) test was used to compare between PTN and HC groups; correlation analysis was performed on clinical data and DTI-related diffusion values of the PTN group; independent samples *t*-test was used to compare the measured values on the affected side of patients between subgroups. All data were analyzed in SPSS 23.0 software, and two-sided *p*-values < 0.05 were considered statistically significant. Linear mixed effects (LME) model was used to explore whether there was significant difference between the pre-operation and post-operation groups of the diffusion values using statistical software (R version 4.0.4).^3^ Due to the small sample size and many measurement indicators, principal coordinates analysis (PCoA) was used to conduct an overall analysis of the differences in the data between the affected side of the PTN group and the HC group. We use ADONIS non-parametric multivariate analysis of variance to test the significance of differences between groups and judge whether the grouping is meaningful. *p* < 0.05 indicates that there is a significant difference between the two groups after PCoA.

### 2.4. Patients surgery and follow-up

11 patients in the PTN group underwent surgery, among them 9 with microvascular decompression (MVD) and 2 with radiofrequency thermocoagulation (RFT). 7 patients (5 with MVD and 2 with RFT) participated in the follow-up of DTI at 6 months after surgery, acquisition procedures and data processing were same as above. VAS score was recorded.

## 3. Results

### 3.1. Subjects’ general clinical information

The general information of the PTN group is shown in the Table 1. All subjects were discontinued medication for more than 48 hours prior to MRI scans. A total of 34 volunteers were included in the HC group (24 females and 10 males, with an average age of 57.7 years). After independent samples *t*-test, there was no significant age difference between the PTN and HC groups (*p* = 0.625). Group gender was not statistically significant (*p* = 1.000). All subjects in the PTN group had an average VAS pain score of 7.19; all subjects in the HC group had an average VAS pain score of 0; the average VAS pain score was 0.27 in all 11 patients who were followed up 6 months after surgery.

**TABLE 1:** Patients’ general information table.

No	Gender	Age (year)	Course of disease (years)	Side	Brunch	Drug (0 = no, 1 = yes)	VAS	Affected side NVC	Unaffected side NVC	Surgery	Post-op VAS
1	F	54	8	L	V3	0	6.4	SCA	N	NA	NA
2	F	52	5	R	V2	0	6.4	N	N	MVD	0
3	F	53	4	L	V3	0	7.1	N	N	NA	NA
4	F	57	3	R	V2, V3	1	5.4	SCA	N	RFT	0
5	M	50	3	R	V2, V3	1	6.8	VA	SCA	MVD	0
6	F	72	3	R	V3	1	9.1	SCA	SCA	MVD	1.0
7	F	75	20	R	V2	1	7.7	N	N	RFT	0.8
8	M	64	3	R	V3	1	8.5	AICA	SCA	MVD	0
9	M	57	2.6	L	V3	1	6.6	SCA	N	MVD	0
10	F	53	6	R	V1, V2, V3	1	7.4	SCA	SCA	MVD	1.2
11	M	60	8	L	V1, V2	1	8.5	VA	SCA	MVD	0
12	F	55	0.15	R	V3	0	5.8	SCA	SCA	NA	NA
13	F	61	1	L	V3	0	7.8	N	AICA	NA	NA
14	F	63	8	R	V2, V3	1	6.3	AICA	N	MVD	0
15	F	44	3	R	V2, V3	0	9.5	N	N	NA	NA
16	F	72	1	R	V2, V3	0	6.6	SCA	N	NA	NA
17	M	66	3	R	V1	1	6.4	SCA	SCA	MVD	0

VA, vertebral artery; SCA, superior cerebellar artery; AICA, anterior inferior cerebellar artery; N, not seen; NA, not appliable.

### 3.2. Quantitative analysis of DTI diffusion values

One-way analysis of variance was used for all data to analyze the differences of DTI measurement diffusion values of the patients’ affected side (TN_Affected), the patients’ unaffected side (TN_Unaffected) and the HC group with SPSS23.0 software. Then we used LSD-*t* test method for pairwise comparison among the three groups to see if there was a significant difference between them.

**FIGURE 2:**
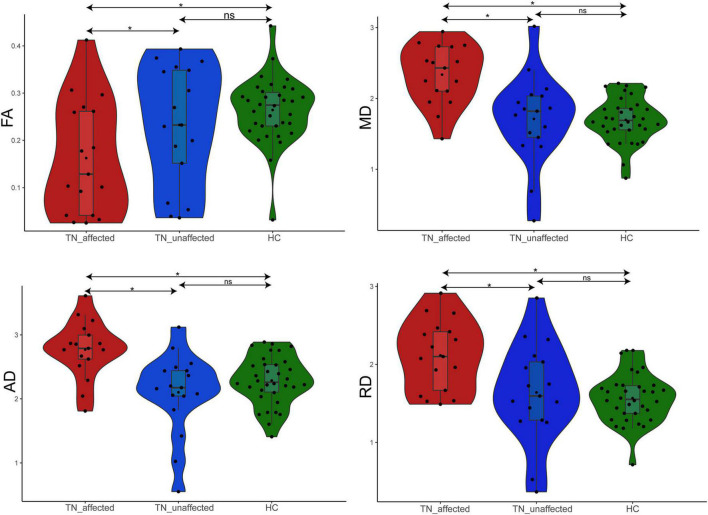
Difference analysis of FA, MD, AD, and RD values in REZ among TN_affected side, TN_unaffected side, and HC groups. *p < 0.05.

The results of the four diffusion values of DTI value measurement on the affected and unaffected side of the PTN group and value of the HC group are shown in Table 2. The results showed: ① FA value: the affected side was 29.7% lower than the unaffected side (*p* = 0.044), 38.1% lower than the HC group (*p* = 0.001), and there was no significant difference between the unaffected side and the HC group (*p* = 0.290). ② MD value: the affected side was 36.6% higher than the unaffected side (*p* < 0.001), 37.6% higher than the HC group (*p* < 0.001), and there was no significant difference between the unaffected side and the HC group (*p* = 0.924). ③ AD value: the affected side was 31.8% higher than the unaffected side (*p* < 0.001), 21.2% higher than the HC group (*p* = 0.001), and there was no significant difference between the unaffected side and the HC group (*p* = 0.190). ④ RD value: the affected side was 28.9% higher than the unaffected side (*p* = 0.003), 34.9% higher than the HC group (*p* < 0.001), and there was no significant difference between the healthy side and the HC group (*p* = 0.579) (Figure 2).

**TABLE 2:** Statistical table of FA, MD, AD, and RD values in REZ of PTN and HC groups.

Group	FA		MD		AD		RD	
	**Mean ± SEM**	**SD**	**Mean ± SEM**	**SD**	**Mean ± SEM**	**SD**	**Mean ± SEM**	**SD**
TN_affected	0.166 ± 0.029	0.120	2.336 ± 0.101	0.415	2.771 ± 0.108	0.445	2.116 ± 0.109	0.449
TN_unaffected	0.236 ± 0.031	0.127	1.710 ± 0.151	0.623	2.102 ± 0.520	0.626	1.641 ± 0.152	0.626
HC	0.268 ± 0.012	0.069	1.698 ± 0.537	0.313	2.286 ± 0.065	0.379	1.568 ± 0.054	0.315

SEM, standard error; SD, standard deviation; TN_affected, the affected side of the PTN group; TN_unaffected, the unaffected side of the PTN group.

### 3.3. Correlation analysis of DTI diffusion values and clinical indicators in PTN group

**FIGURE 3:**
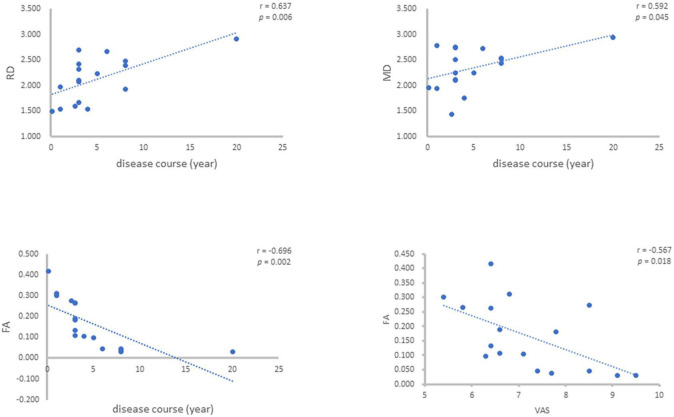
Correlation between FA, MD, AD, and RD of PTN patients and their disease course and VAS.

For the values of FA, MD, AD and RD of the patients’ affected side measured above, the correlation analysis was carried out with the disease course (years) and VAS of the patients, and it was concluded that the FA value was negatively correlated with the disease course (*r* = –0.696, *p* = 0.002), MD value was positively correlated with disease course (*r* = 0.492, *p* = 0.045), AD value had no correlation with disease course and RD value was positively correlated with disease course (*r* = 0.637, *p* = 0.006). FA value was negatively correlated with VAS (*r* = –0.567, *p* = 0.018), MD, AD and RD values were not correlated with VAS (Figure 3).

### 3.4. Difference analysis of DTI diffusion values and clinical indicators in PTN group

The PTN group were divided into two groups whether there was vascular nerve compression (NVC) on the affected side or not, and the independent samples t-test was used to analyze whether the four diffusion values of DTI were different between the two groups. It was concluded there was no significant difference in diffusion values between the NVC group and the non-NVC group. Similarly, PTN patients were divided into two groups based on whether they had a history of carbamazepine and related drugs. The result showed there was no significant difference in diffusion values between the medication group and the non-medication group.

### 3.5. Difference analysis of DTI diffusion values of pre-operation and post-operation in PTN group

The DTI-related diffusion values of 7 surgical patients before and 6 months after operation were compared. The diffusion values of the four voxels in the REZ area before and after the operation of the 7 PTN patients were measured respectively in the affected side, and a total of 16 diffusion values (FA, MD, AD and RD each include four values) were obtained for each side. Group analyses were performed using a linear mixed effects model including the pre-operation and post-operation groups with LME. The result showed significant difference for four DTI-related diffusion values at 6 months after operation. FA increased from baseline of pre-operation [*F*(48.000) = 78.867, *p* = 1.063 × 10^–11^], AD decreased from baseline of pre-operation [*F*(48.000) = 210.99, *p* < 2.200 × 10^–16^], RD decreased from baseline of pre-operation [*F*(48.000) = 178.04, *p* < 2.200 × 10^–16^], MD decreased from baseline of pre-operation [*F*(48.000) = 282.24, *p* < 2.200 × 10^–16^]. These trends were restored to the values of the HC group. Therefore, it was concluded that postoperative DTI-related diffusion values of patients with trigeminal neuralgia tended to change to healthy controls.

### 3.6. Principal coordinate analysis (PCoA) of DTI comprehensive indexes among the affected side of PTN group, the unaffected side of PTN group and HC group

**FIGURE 4:**
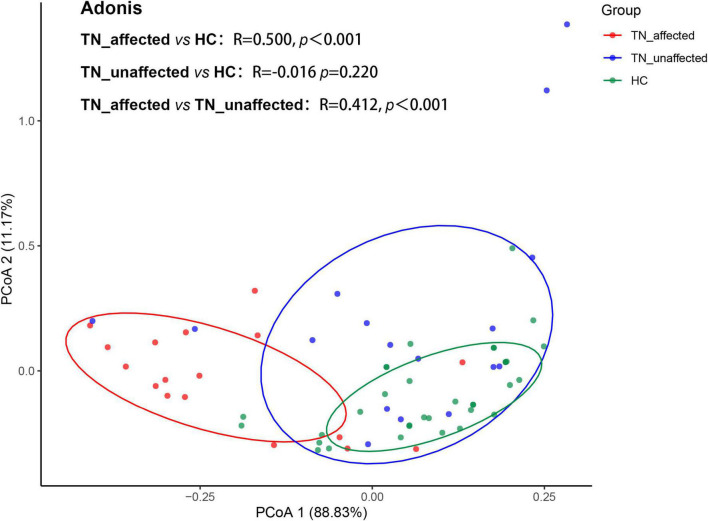
Principal coordinates analysis (PCoA) of the affected side (TN_affected), unaffected side (TN_unaffected) in the PTN group and HC group showed significant differences in the four DTI indicators between TN_affected and HC groups (*r* = 0.500, *p* < 0.001) and TN_affected and TN_unffected groups (*r* = 0.412, *p* < 0.001), but no significant differences between TN_unaffected and HC groups (*r* = –0.016, *p* = 0.220).

PCoA could reduce dimensionality and perform similarity or difference analysis on data sets to simplify the data. The principal coordinate analysis is based on the Bray-Curtis distance. Each point in the figure represents a subject (red represents subjects in the PTN group, and green represents subjects in the HC group). According to the diagram, when the distance between the two points is closer, it means that the difference is smaller, and vice versa, it means that the difference between the two groups is larger. In this trial, Adonis test was performed on the difference of all four comprehensive indexes of DTI between the affected side of PTN group and HC group, the affected side of PTN group and unaffected side of PTN group, the unaffected side of PTN group and HC group. The results showed that there was significant difference in the overall index between the affected side of the PTN group and the HC group (*r* = 0.500,*p* < 0.001), between the affected side and the unaffected side of the PTN group (*r* = 0.412,*p* < 0.001), and there was no significant difference between unaffected side of the PTN group and the HC group (*r* = –0.016,*p* = 0.220), so it was concluded that DTI-related diffusion values had the potential to become an objective auxiliary evaluation of PTN diagnosis or treatment criteria (Figure 4).

## 4. Discussion

The trigeminal nerve is the fifth cranial nerves. PTN refers to the recurrent sudden, severe, lightning-like tingling pain in the distribution area of the trigeminal nerve. Secondary neuralgia symptoms due to various other central nervous system diseases must also be excluded, such as multiple sclerosis and intracranial space-occupying lesions. At present, little is known about the etiology of trigeminal neuralgia. The current world-wide theory is vascular compression proximal to the REZ of the trigeminal nerve – NVC (Jannetta, 1967). The distance between the REZ area and the brain stem is about 2–6 mm (Maarbjerg et al., 2015). REZ area is the transition zone between the central nervous system and the myelin sheath of the peripheral nervous system, which is considered to be most susceptible to vascular compression (Woolfall and Coulthard, 2001). The NVC leads to local microstructural changes in the REZ area, and secondary demyelination of the trigeminal nerve root (Liu et al., 2013). Abnormal remyelination of the demyelinating zone and formation of ectopic synapses due to repeated minimally invasive trauma, possibly related to vascular pulsation, allow sensory transmission through ectopic pacemaker and tactile cross-linking and cross-discharge between axons to make sensory input extremely excited (Devor et al., 2002). Ordinary trigeminal nerve-related magnetic resonance scanning technology can generally only show the approximate running direction of the trigeminal nerve in the cerebellopontine angle and whether there is vascular compression around. The extent to which the fiber bundles were deviated, and the nerve was compressed or deformed was not quantified.

DTI technology has been used since 1996 and has been widely used in recent years. It can be analyzed from different angles through the scanning of multiple gradient fields, and the data of the diffused signals in all directions can be quantified (Fujiwara et al., 2011; Neetu et al., 2016). As a non-invasive MRI method, DTI is increasingly used in the study of neurological or brain diseases. After peripheral nerve injury or sustaining chronic pain, abnormalities in the white matter of the brain will occur (Rappaport and Devor, 1994; Davis et al., 2011). DTI not only has certain reference value and significance for the course of white matter fibers, but also evaluates the integrity of the tissue structure (Leal et al., 2011). This technique has been applied to PTN research (Herweh et al., 2007; Upadhyay et al., 2008; Hodaie et al., 2012). Microstructural abnormalities in the REZ region in PTN patients with NVC has been verified (Van Der Molen, 2018). There are four main diffusion values of DTI, including FA (providing information on the integrity of fiber bundles), MD (reflecting the level of diffusion and diffusion resistance), MD (reflecting the level of diffusion and diffusion resistance), AD (reflecting the local integrity of axons; radial diffusion ratio, and RD (reflecting pathological changes such as demyelinating changes, neuroinflammation and edema of nerves) (Aung et al., 2013).

By measuring diffusion values, previous studies (Song et al., 2003; Leal et al., 2011) found that FA decreased in the REZ of the trigeminal nerve while MD increased in the REZ. Leal et al. (2011) studied the DTI parameters of TGN in 10 TN patients with NVC and found that compared with the control group, the FA value of the affected side of TGN was significantly lower and the AD value was significantly higher. However, other parameters have not been studied. In most of DTI related research in PTN, there was few correlations analysis between the values with clinical manifestations.

The FA value is usually used to describe the integrity of nerve fibers, and the MD value reflects the diffusion resistance and the molecular weight of water in the tissue. Long-term NVC will lead to axonal demyelination and degeneration at first, and FA value decreased. The loss of tissue structure in the REZ, in turn, leads to a decrease in diffusion barriers and an increase in MD values, so a decrease in the FA value on the affected side is negatively correlated with an increase in the MD value (Leal et al., 2011). Nerve atrophy is considered a late consequence of chronic physiological stress after demyelination (Gizewski et al., 2014). Low FA and high MD suggested pathological features of axonal demyelination, neuroinflammation and edema, and so on. These results are consistent with the pathological manifestations of axonal loss, axonopathy, demyelination, abnormal demyelination, residual myelin debris, and collagen deposition in nerve specimens from PTN patients.

Among the 17 patients included in this study, 12/17 had NVC manifestations of the affected side on MRTA, and 8/17 had NVC manifestations on the patients’ healthy side. The results of diffusion values analysis showed that, regardless of whether there was vascular compression, the overall mean of the patients’ healthy side of PTN group was not significantly different from that of the HC group; the DTI parameters of the patients’ affected side were significantly different from those of the healthy side of PTN group and the HC group; the DTI parameters have no statistical difference between the NVC group and None-NVC group. These results indicate that there is no correlation between the occurrence of microstructural changes in the REZ of the trigeminal nerve in PTN patients and NVC, which needs to be further verified by expanding the sample size.

The results of this study showed that the increase of the duration of PTN was related to the decrease of the FA value and the increase of the MD and RD values of the patients, which suggesting that the duration of pain was an important factor affecting the microstructure changes of the trigeminal nerve. The correlation analysis between the patients’ DTI parameters and the VAS pain score showed that the FA value was negatively correlated with the VAS, indicating that the more severe the microstructure change of the trigeminal nerve fiber, the more severe the pain. At the same time, it was observed that there was no significant difference between the medication history and the changes of DTI parameters in the REZ. In this study, the PCoA between the PTN group and the HC group was carried out for all indicators, and there were significant differences between the affected side of the PTN group and the HC group.

Among the 17 patients, 11 patients underwent surgical treatment, of which 9 patients underwent MVD surgery, and 2 patients underwent RFT. The symptoms of all patients were relieved after surgery, and 7 patients participated in DTI 6 months after surgery. The follow-up data showed that among the DTI diffusion values of the 7 patients, the FA value showed an upward trend after surgery, and the MD, AD and RD values all showed a downward trend. Postoperative DTI-related diffusion values of patients tended to change to healthy controls. It is suggested that the relief of symptoms in patients with PTN contributes to the improvement of microstructural abnormalities in the REZ of the trigeminal nerve.

There are still some shortcomings in this trial. For example, the sample size is not large enough, which leads to some problems such as inconsistency between some negative conclusions and positive results reported in the literature. It needs to be supplemented with relevant data in the future to improve. The connection and changes of the peripheral structure and the central structure of the trigeminal nerve in the pathogenesis of PTN need to be further confirmed by follow-up studies.

## Data availability statement

The original contributions presented in this study are included in the article/Supplementary material, further inquiries can be directed to the corresponding authors.

## Ethics statement

The studies involving human participants were reviewed and approved by the Ethics Committee of the Ninth People’s Hospital Affiliated to Shanghai Jiao Tong University School of Medicine. The patients/participants provided their written informed consent to participate in this study.

## Author contributions

YWa designed the study, collected the data, and wrote the manuscript. DW performed the imaging data. YWu coordinated issues related to imaging tests. CZ provided guidance and assistance to statistics. WW and LL collected the clinical data of all subjects. YL adjusted imaging parameters and guided image processing. MC and WC provided test and manuscript writing ideas and subsequent revision.

## Footnotes

1
www.fmrib.ox.ac.uk/fsl

2
http://dsi-studio.labsolver.org/download.html

3
http://www.r-project.org/
