# Supplementary material for: A preliminary study of diffusion tensor imaging in root entry zone of primary trigeminal neuralgia
Source: Front Neuroanat. 2023 Mar 22;17:1112662. doi: 10.3389/fnana.2023.1112662 (PMC10073458; doi:10.3389/fnana.2023.1112662)
Supplement: Supplementary file 1 [file Data_Sheet_1.docx]

**Supplementary material**


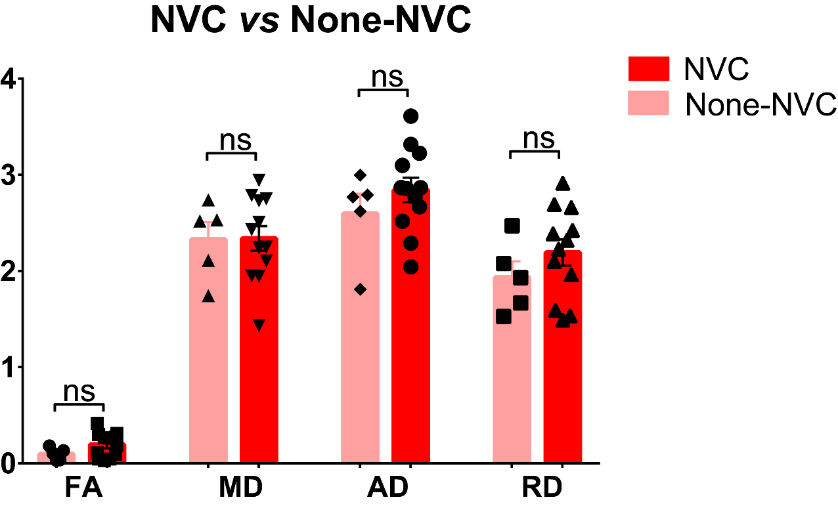


Fig.2-1 Difference analysis of FA, MD, AD and RD values between NVC and None-NVC group in PTN patients. There was no significant difference in DTI-related parameters between two groups.


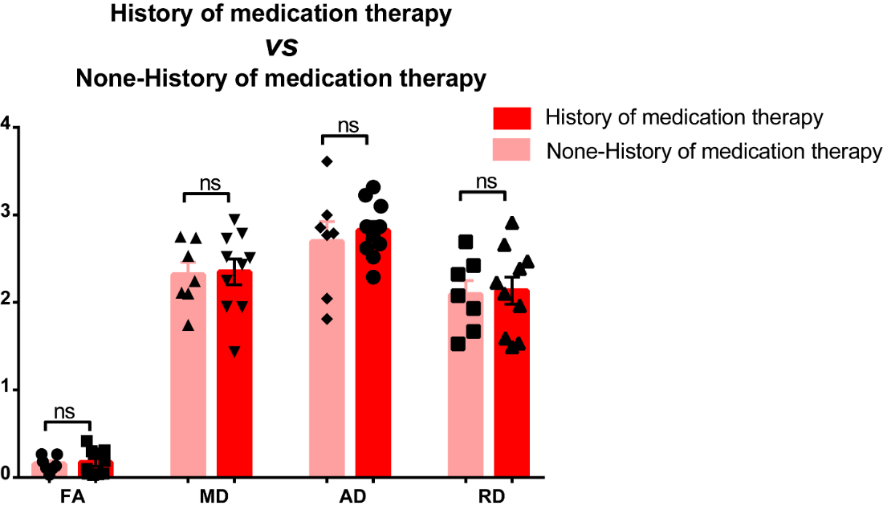


Fig.2-2 Difference analysis of FA, MD, AD and RD values between PTN patients with and without medication history. There was no significant difference in DTI-related parameters between two groups.
